# 

**Published:** 1896-05-09

**Authors:** 


**Notices of Births, Deaths, and Marriages** are inserted in this column at a charge of 2s. 6d. for an announcement not exceeding four lines.

### NOTICE TO CORRESPONDENTS.

All MS., Letters, Books for Review, and other matters intended for the Editor should be addressed The Editor, The Lodge, Porchester Square, London, W.

The Editor cannot undertake to return rejected MS., even when accompanied by stamped directed envelope.

**Notice to Advertisers and Subscribers.**

All Advertisements, Orders for Copies of The Hospital and Business Communications should be addressed to **THE MANAGER, (Not to The Editor),** The Hospital, 428, Strand, London, W.C.

The Cost of Subscription, post free, is as follows:—Per week, 2½d.; per quarter, 2s. 8d.; per half-year, 5s. 3d.; per annum, 10s. 6d. Foreign:—Per Annum, 15s. 2d.; payable, in all cases, in advance.

Ready Shortly.

**"Burdett's Hospitals and Charities, 1896."**

Seventh year of publication. Over 1,000 pp. Scarlet cloth, gilt lettered. Price 5s. A most complete guide to British, Colonial, Indian, and American Hospitals, Dispensaries, Nursing and Convalescent Institutions, and Asylums. A few complete sets of the preceding six issues of the "Annual" may still be obtained on application to the Publishers, The Scientific Press (Limited), 428, Strand, London, W.C.

**NOTICE.—Vol. XIX. of "The Hospital" (October, 1895, to March, 1896), in handsome cloth binding, is now ready and on sale at the Office of this Journal. Price 7s. 6d. Cases for binding the Numbers contained in this Volume, 1s. 6d. Index to Vol. XIX., 2½d., post free.**

**The Monthly Part for May is now ready, price 6d., by post, 8d.**

### The Student of Medicine.

### APPOINTMENTS.

J. Anderson, C.I.E., F.R.C.P., appointed Lecturer on Tropical Diseases at St. Mary's Hospital Medical School. J. C. H. Beaumont, L.R.C.P., L.R.C.S.Edin., appointed Medical Officer to the Infirmary and the Gordon Road Workhouse of the Camberwell Parish. D. J. S. Burt, M.B., C.M.Edin., appointed House Surgeon to the Brighton, Hove, and Sussex Throat and Ear Hospital, Brighton. R. L. Clark, M.B., C.M.Edin., appointed Medical Officer for the Maryport District of the Cockermouth Union. R. B. Duncan, M.D.Durh., appointed Medical Officer for the Bradninch District of the Tiverton Union. H. A. Edmonds, M.R.C.S., L.R.C.P.Lond., appointed Medical Officer for the Nettlebed District of the Henley Union. G. B. Flux, M.D.Brux, L.R.C.P., M.R.C.S., L.S.A., appointed Anæsthetist to the National Dental Hospital. R. A. Flynn, F.R.C.P., L.R.C.S.I., appointed Gynæcologist to the Drumcondra Hospital, Dublin. T. Garstang, M.R.C.S.Eng., appointed Medical Officer of Health to the Middlewick Urban District Council. S. E. Gill, M.B.Lond., L.R.C.P., M.R.C.S., appointed Resident Medical Officer to the Royal Hospital for Diseases of the Chest, City Road. H. T. Jones, M.R.C.S., L.R.C.P.Lond., appointed Medical Officer for the Second District of the Pembroke Union. S. E. Jones, L.R.C.P., L.R.C.S., appointed Medical Officer for the Shocklack District of the Tarvin Union. C. E. Lansdown, M.R.C.S., L.R.C.P.Lond., appointed Resident Medical Officer to the Royal York Dispensary. B. J. Macaulay, L.R.C.P., L.R.C.S.Edin., L.F.P.&S.Glasg., appointed Clinical Assistant to the Hospital for Consumption and Diseases of the Chest, Brompton. Dr. J. Mackenzie, appointed Medical Officer for the Kirkby District of the Basford Union. J. D. R. Munro, M.D.Edin., appointed Medical Officer of Health to the Nantwich Urban District. R.S. Pearson, M.R.C.S., L.R.C.P., appointed Medical Officer of Health for the Wigan Rural District. H. G. Plimmer, M.R.C.S., L.S.A., appointed Bacteriologist at St. Mary's Hospital Medical School. W. Scatterty, M.A., M.D.Aberd., appointed Medical Officer of Health for the Borough of Keighley. H. Walsham, M.A., M.B.Cantab., M.R.C.P.Lond., appointed Assistant Physician to the City of London Hospital. W. W. Williamson, M.B., C.M.Edin., appointed Medical Officer of Health to the Lynn Urban District.

### VACANCIES.

The following vacancies are announced this week, the amount of salary in each case being given after the name of the institution:—

### Resident Medical Officers.

Buluwayo Hospital.—Resident House Surgeon; unmarried. Salary £400 per annum, with free board and lodging. Applications to be addressed to "The Chairman, Hospital Board, Buluwayo, Rhodesia," to reach not later than June 30th.

Burgh of Leith.—Resident Physician to the Public Health Hospital; doubly qualified. Salary 50 guineas. Applications to T. B. Laing, Town Clerk, Town Clerk's Office, Leith, by May 9th.

Donegal District Lunatic Asylum, Letterkenny.—Assistant Medical Officer; qualified in medicine, surgery, and midwifery; unmarried, and not more than 30 years old. Salary £100 per annum, with furnished apartments, rations, washing, fuel, light, and attendance, valued at £100 per annum. Applications to Dr. Moore, Resident Medical Superintendent, by May 9th.

Glamorgan County Asylum, Bridgend.—Junior Assistant Medical Officer. Salary £130, rising £10 a year to £150 per annum, if approved, with board (no beer or wine), lodging, and washing. Applications to the Medical Superintendent by May 12th.

Hospital for Women, Soho Square, W.—House Physician; doubly qualified. Appointment for six months. Salary £30. Applications to David Cannon, Secretary, by May 9th.

Liverpool Infirmary for Children, Myrtle Street, Liverpool.—House Surgeon. Salary £85 per annum, with board and lodging. Applications to C. W. Carver, Honorary Secretary, by May 9th.

National Hospital for Diseases of the Heart and Paralysis, Soho Square.—Resident Medical Officer; doubly qualified. Appointment for six months. Board, residence, laundry, and honorarium of 10 guineas. Applications to the Secretary by May 11th.

Royal South Hants Infirmary, Southampton.—Assistant House Surgeon. Appointment for six months. Gratuity £10. Applications to T. A. Fisher Hall, Secretary, by May 15th.

Victoria Hospital for Sick Children, Queen's Road, Chelsea, S.W.—House Physician for In-patients. Honorarium £50 per annum, with board and lodging. Appointment for twelve months. Assistant Physician to Out-patients. Appointment for five years, but eligible for re-election. Applications to the Secretary by May 16th.

### Non-Resident Medical Officers.

Dental Hospital of London, Leicester Square. W.—Anæsthetist and Assistant Anæsthetist. Must be duly qualified medical practitioners. Applications to J. Francis Pink, Secretary, by May 11th.

Dental Hospital of London and London School of Dental Surgery, Leicester Square.—Lecturer on Mechanical Dentistry. Applications to Morton Smale, Dean, by May 11th.

Plymouth Royal Eye Infirmary.—Honorary Surgeon. Applications to the Secretary by May 9th.

